# Tea and Coffee

**Published:** 1870-02

**Authors:** 


### TEA AND COFFEE,

Used at each regular meal, as the exclusive drink of all classes and all ages, will add to the health, life, happiness, and well-being of any nation. All nations, of all ages, have solid or liquid excitants, or stimulants, made to hand, or have discovered or invented them, or found out the mode of use adapted to the results. It would seem from this, that a beneficent Providence intended their employment for the comfort of the creatures of his power; written revelation giving the explicit conditions of their use.

"TEMPERANCE."

The conditions of age and sickness and necessary toil, demand, imperatively demand the use of stimulants, or excitants, at certain times. The wrong is in the abuse, and not the temperate use of these things. In civilized society there are a thousand dyspeptics where there is one drunkard; a dyspeptic is not made by the use of meat and bread, but by the abuse of it.

We are speaking now of natural stimulants and excitants, which are made to our hand—red pepper, for example. Alcoholic preparations are not made to our hand; they are not natural; they are the contrivances of human ingenuity, and are left out of consideration altogether; none of them are necessary to human health or comfort, at least, under the ordinary circumstances of man's condition on earth; and a happy thing would it be for all mankind in the extended average of human life and in general, material, and moral thrift, if not another drop of alcohol were manufactured or another grain of opium were produced until the end of time.

It is in connection with the table that a vast amount of our drunkards are made; this host would be cut off at once if only tea and coffee were drank.

COLD WATER,

Or any other cold drink, taken at meals, largely and habitually, will inevitably produce dyspepsia, in all who live mostly indoors; for educated medical men have seen with the eyes, that if a man drinks any thing cold while eating, or soon after, the process of digestion is instantly arrested, and does not begin to proceed until what was drank is raised to the natural heat of the body, which, in round numbers, is one hundred degrees Fahrenheit. The common ice-water of our tables is about forty degrees, good spring water is not so cool; but it will be seen that to warm a glass of ice-water from forty to a hundred degrees requires considerable heat. As soon as it is swallowed, nature seems to take the alarm, and by her instincts sends or spares heat from other parts of the body to warm the water; but, in doing so, robs them of their healthful amount; the result being, perhaps in the reader's own experience, that the person rises from the dinner-table with little chills running over him—along the back, or the hands and feet are cold. Medical books record cases of congestive chills, pneumonia, and immediate death from this very cause. But moderate injuries of this kind, repeated every day, tell eventually, and leave the patient dyspeptic for life, or with so little vitality as to be a permanent invalid.

### "CAN'T STAND ANY THING."

As a general rule, the foundations of consumption and dyspepsia are laid before twenty; hence if children were required to drink something hot, or, at least, very warm, one important cause of the two common diseases named would be removed. We naturally revolt at the idea of drinking hot water, and probably would never get used to it, hence it would seem that something should be added to the water to make it palatable, and if this added ingredient is prepared to our hand by nature, and has beneficial properties of its own, it would seem proper and right to use it. Both tea and coffee are found to answer these conditions; one found in the eastern hemisphere, the other in the western; one the product of northern climes, the other of southern; and both containing the same identical ultimate ingredients. One adapted to bilious temperaments, the other to those which are not bilious; for coffee does make some persons bilious, or causes other undesirable symptoms, such as sleepiness, headache, languor, heartburn, or the like. Of course, persons thus affected should not use it, but should employ tea; on the other hand, if tea causes disagreeable sensations in others, common sense indicates that it should not be used.

Every one knows what a prompt and delightful condition is induced, when tired, and cold, and hungry, by taking a bowl of hot, well seasoned soup, but not if the soup were cold; this proves that the element of heat is essential to a healthful meal. Some of the most terrible of all diseases are induced by eating cold food habitually. Hence, tea and coffee should be used hot, and it would be wicked, at least for the old, and frail, and feeble, and overworked, not to use them at meals when they can get them. The essential element of tea and coffee has the inevitable effect on the human system of enlivening, of invigorating both mind and body for the time, and thus, when weary and hungry, elevates the whole man to the condition of receiving and managing the food eaten; that is, promotes digestion thereby; hence tea and coffee at our meals are

GOOD DIGESTERS.

For these reasons their habitual use is advocated at every meal, to the exclusion of cold water, sweet milk, wine, beer, and brandies.

The use of milk and sugar with coffee and tea arose from a desire originally to make them palatable, just as sirup and other sweets are employed to make medicines palatable; but they not only do not add to the native virtues and benefits of these drinks, but dilute, injure, and destroy. Whatever benefit is derived from tea and coffee, is lessened by the addition of any other ingredient; we dilute them too much with water, and digestion itself is injured by taking a large quantity of any kind of liquid into the stomach; one ordinary sized teacupful is enough for the usual meal, but it should be always hot; if only one cup be used at a meal, and the strength of it slowly increased in the progress of years, tea and coffee will be an increasing, delightful, and healthful beverage to the end of life.

If, now and then, a person finds that his system does not tolerate them, it is because of some peculiarity, and some other warm drink should be taken—sassafras or any of our garden herbs. Such a course will prevent innumerable cases of dyspepsia, and will save many a person from falling into habits of intemperance.

